# The Periods at Which Tetanus Occurs

**Published:** 1846-05

**Authors:** 


*The Periods at which Tetanus occurs.*—If it sets in soon after the injury that caused it, it will run its course rapidly. Thus, suppose a man gets a hurt in the morning, and in the evening of the same day tetanus appears; he dies that night. Whenever it comes on so soon as this, the patient invariably dies in twenty-four hours; but you may not meet with such a case as this in twenty years. In these countries tetanus seldom makes its appearance before the sixth day after the injury; it sometimes will not show itself until the forty-second day. The wound may be in any possible state or stage; it may be sloughing, or the slough may have separated, or more generally when the wound is suppurating, and as I have myself seen, even when the wound is quite healed. There is, then, no stage of the local injury in which tetanus may not make its appearance; and neither before nor during the progress of the complaint does the wound alter its character in the least, as it does in hydrophobia.—*Professor Colles' Lectures.*

